# The contribution of leaders' and managers' attributes, values, principles, and behaviours to the sustainable implementation of Lean in healthcare: A realist review protocol

**DOI:** 10.12688/hrbopenres.13933.1

**Published:** 2024-08-13

**Authors:** Anne Marie Keown, Seán Paul Teeling, Martin McNamara

**Affiliations:** 1 UCD Centre for Interdisciplinary Research, Education and Innovation in Health Systems, School of Nursing, Midwifery & Health Systems, University College Dublin, Dublin, D04 V1W8, Ireland; 2 Centre for Person-Centered Practice Research,Division of Nursing, School of Health Sciences, Queen Margaret University Drive, Queen Margaret University, EH21 6UU Musselburgh, UK

**Keywords:** Realist Review, Protocol, Leadership, Lean, Improvement, Healthcare.

## Abstract

**Background:**

Global healthcare faces challenges such as rising costs, budget constraints, aging populations, chronic diseases, and increasing patient expectations. Healthcare organisations are deploying continuous improvement methodologies to address these challenges. Lean, derived from the Toyota Production System, focuses on eliminating non-value-adding activity and enhancing efficiency, making it a prominent quality improvement approach in healthcare. Effective implementation of Lean requires robust leadership to sustain improvements and foster a culture of continuous improvement. However, the attributes, values, principles, and behaviours of effective Lean leaders in healthcare remain underexplored.

**Methods:**

This realist review protocol details methods to research how leaders' and managers' attributes, values, principles, and behaviours contribute to the sustainable implementation of Lean in healthcare. Following the RAMESES guidelines, a five-stage structured methodology will be used: defining the scope of the review and developing initial theories, developing the search strategy, reviewing primary studies and extracting data, synthesising evidence and developing conclusions, refining theory iteratively, and disseminating findings. An Expert Panel and reference groups of healthcare managers and leaders will refine candidate programme theories (CPTs) into initial programme theories (IPTs), guiding detailed evidence searches and data extraction.

**Conclusion:**

This realist review will deepen our understanding of the specific mechanisms by which leadership impacts Lean implementation outcomes in the context of acute hospitals. By exploring how leadership attributes, values, principles and behaviours shape outcomes for diverse stakeholders, the review aims to provide critical insights into the dynamics driving the success of Lean in healthcare. The findings will inform policy and practice, enhancing leadership strategies to improve patient and staff experiences, patient outcomes, and organizational performance.

## Introduction

Global healthcare is experiencing significant disruption due to rising care costs, budget constraints, ageing populations, chronic diseases, and increasing patient expectations. These challenges necessitate innovative approaches to improve performance ^1– 3^. It has been argued that this requires new leadership and management strategies ^1^. An analysis of 30 years of service reorganisation in Ireland, concluded that effective and sustainable change requires not only incremental improvements but also innovative transformations in care delivery and leadership practices ^4^. Recent studies ^4– 6^ highlight that traditional leadership approaches are insufficient for the complexities of modern healthcare.

### Continuous improvement

In response to these complexities, Quality Improvement (QI) methodologies like Lean, Six Sigma, and Lean Six Sigma (LSS) have been used for continuous improvement and have shown to be effective for enhancing organisational efficiency and ensuring high-quality patient care ^7, 8^. A survey by the University of California, Berkeley found nearly 70% of US healthcare systems engaged in improvement initiatives such as Lean thinking ^1^. However, only 12% of hospitals reported mature, hospital-wide implementation, highlighting the need for leadership commitment and daily management systems ^1^.

Aligning improvement methodologies with health system priorities and developing a shared culture of problem-solving, driven by leadership commitment has been promoted ^9^. Lean has emerged as a globally recognised management improvement paradigm, extending beyond manufacturing to various sectors, including healthcare ^10^.

### Lean in healthcare

Lean, derived from the Toyota Production System (TPS), focuses on eliminating activities that do not add value to a process to improve the flow of people, information, or goods ^11, 12^. Initially popularised by Womack ^13^, Lean aims to increase care quality and efficiency ^14^. It has become a dominant quality improvement approach in healthcare literature, emphasising the need for an organisational strategy that integrates Lean principles, culture, and focused leadership ^15^. Effective Lean implementation in healthcare requires system-wide change and strong leadership to sustain improvements ^16^. Leadership is crucial for creating a continuous improvement culture, empowering frontline staff, and aligning organisational values and behaviours with Lean principles ^12, 17^.

### Lean leadership

Lean leadership is essential for the sustainable integration of Lean in healthcare, focusing on values, respect, consensus on behaviours, and a no-blame culture ^18^. Successful Lean leaders foster a culture of collaboration, problem-solving, and continuous improvement ^19^. Despite its importance, the specific attributes and behaviours of effective Lean leaders in healthcare are not well-defined in the literature ^20– 22^. Understanding the attributes, behaviours, values, and principles of Lean leadership is crucial for effective Lean deployment and strategic healthcare management. This protocol paper outlines a realist review of literature that forms part of a wider realist inquiry that aims to explore these aspects, addressing the gap in scholarly understanding of Lean leadership in healthcare.

### Use of realist inquiry

Given the low maturity of Lean leadership as a scientific topic, literature reviews are scarce and often treat it as a secondary theme ^23^. The risk of attributing causal power to healthcare leadership for the outcomes of Lean systems without clarity on the constructs and causal mechanisms involved has been highlighted ^19^. Current definitions of Lean leadership lack a theoretical basis related to general leadership theories, leading to unclear rationales and divergent aspects. Existing research has not addressed the causal mechanisms by which leadership behaviours influence Lean implementation ^19, 24^. The complexity and context-dependence of Lean leadership behaviours in healthcare make this a subject well-suited to realist inquiry.

Pawson argues that realism serves as a methodological framework guiding various research methods ^25^. Bhaskar's work positions realist inquiry within the broader realist philosophy of science and social science, emphasising the identification of generative causation through underlying causal mechanisms ^26^. Realist inquiry aims to understand "what works, for whom, under what circumstances, and why" ^12, 25, 27, 28^. Realist inquiry comprises realist review and realist evaluation ^25^. Realist review (or synthesis) examines existing data or stakeholder perspectives, while realist evaluation involves primary research with direct data collection. This approach has been widely used in healthcare and is growing in popularity for evaluating Lean healthcare interventions ^12, 28, 29^.

Realist inquiry goes beyond exploring surface-level inputs and outputs by discerning how the resources of a specific intervention or programme evoke and interact with stakeholders’ psychosocial responses, and mechanisms (M) that trigger intervention outcomes (O) in specific contexts (C) ^30^. Researchers develop a Context-Mechanism-Outcome (CMO) hypothesis to predict which mechanisms operate in different contexts and the resulting outcomes ^31^. This configuration is ideal for exploring the context-sensitivity of leaders’ behaviours and the contextual factors impacting Lean implementation in healthcare. It can be applied to Lean leadership in specific contexts (C) to investigate what resources it provides and what responses these resources evoke (M), that generate observed outcomes (O).

## Protocol

The realist review outlined in this protocol seeks to understand how and under what circumstances the attributes, values, principles, and behaviours of leaders and managers contribute to the sustainable implementation of Lean in healthcare settings. This review will explore the specific mechanisms by which leaders' practices, impact implementation outcomes both anticipated and unanticipated in various contexts. Leadership behaviours may affect different groups, including frontline staff, middle managers, and senior executives, in distinct ways ^32^. By investigating how leadership attributes and behaviours shape outcomes for these diverse stakeholders, this review aims to deepen our understanding of the dynamics that drive the success of Lean implementation in healthcare. In doing so, it will offer critical insights into how leadership and contextual factors interact to influence the effectiveness of Lean methodologies.

The research question guiding the realist review described in this protocol is outlined below:

•   In acute hospital settings where staff are trained in Lean and it is implemented as an improvement methodology, what are the specific attributes, values, principles, and behaviours of Lean leaders and managers, and how do the mechanisms these activate along a continuum for different stakeholder groups impact the anticipated outcome of sustainable Lean deployment?

### Methods

A realist review interprets existing data through a realist approach applied to retrospective literature reviews ^33^. This method acknowledges that theories may not always predict outcomes across all contexts ^34^. Realist reviews have been described as explanatory and non-judgmental, iteratively testing and building theory ^35^. The RAMESES guidelines provide standards to improve the rigor and comparability of realist and meta-narrative reviews ^36^. A five-stage structured methodology consistent with the RAMESES guidelines has been developed ^37^ which we have utilised to inform this review:

1. Define the scope of the review and develop initial theories.

2. Develop the search strategy.

3. Review primary studies and extract data.

4. Synthesise evidence and develop conclusions.

5. Refine theory iteratively and disseminate findings.

***1. Define the scope of the review and develop initial theories***

The development of candidate programme theories (CPTs) in a realist review provides a framework for analysing interventions' causal mechanisms that generate the outcome of an intervention in specific contexts ^28^. Developing CPTs involves mapping out potential explanations of how the intervention works. This theoretical mapping typically draws on a scoping review of existing literature, expert insights, and preliminary data, forming a hypothesis about the intervention’s mechanisms ^38^. 

The realist review described in this protocol focuses on theories explaining how leadership behaviours and attributes influence the sustainability of Lean implementation in healthcare. The identification of relevant theories for the realist review began with a preliminary scoping search. A preliminary background search in key databases was undertaken searching by titles of articles, abstracts, keywords, and subject headings to guide the development of the CPTs. Consultation with a university librarian enhanced the strategy, ensuring both breadth and depth in the search ^39^ and leading to the use of the largest medical and nursing databases, (Medline, EMBASE, Emerald, EBSCO, CINAHL). The searches included combinations of the keywords (Lean, Six Sigma, Leadership, healthcare, behaviours, attributes, and implementation). Searches yielded a number of articles (n=24) directly and fully related to the research question which contributed to the development of CPT. The initial scoping search, while not exhaustive, was foundational to constructing the review through the development of candidate programme theories (CPTs), crucial for understanding complex interventions.

As CPT are in effect ‘rough’ theories, they provide a beginning framework that is expected to evolve as evidence is gathered and analysed ^40^. Consultations with reference groups of healthcare managers and leaders who spearheaded Lean deployment further informed the development of CPTs for this study. These consultations sought perspectives on contexts, mechanisms, and outcomes of sustainable Lean deployments. Combined with the scoping literature search and team discussions, this developed six CPTs for expert panel review (additional material 1).

Expert panels, comprising practitioners, researchers, and theorists, refine programme theories, ensuring alignment with theoretical expectations and practical realities through rigorous scrutiny and cross-disciplinary validation ^41^. They guide researchers to key literature, assess evidence quality, and interpret complex data, enhancing the review's credibility and acceptance. For this review, an expert panel of Lean experts from academia and practice in Europe, the United States, and Australia has been convened.

***2. Develop the search strategy***

Consistent with realist methodology, the expert panel will confirm, refute, or refine the 6 CPT ^40^ and identify what, in their experience, facilitates or hinders the effectiveness of the intervention (in this case Lean deployment) to deliver anticipated outcomes ^42^. This will further develop the CPTs to Initial Programme Theories (IPTs) which congruent with realist methods, will inform a detailed evidence search based on keywords from these IPTs ^43^. Developing an effective search strategy is crucial for a comprehensive realist review, capturing a broad evidence base to uncover complex phenomena through mechanisms and contextual factors. Initially, broad search terms derived from the IPTs will be used, aligning with PRISMA guidelines for systematic reviews ^44^ and RAMESES guidelines for realist reviews ^35^.

The search will use multiple sources, supported by a librarian, including internet search engines, electronic databases, and literature citing the included papers, references within those papers, relevant grey literature, and organizational websites. Suggestions from the expert panel will also be incorporated to ensure comprehensive coverage. The inclusion and exclusion criteria for the literature review will be systematically outlined using the PICOC (Population, Intervention, Comparison, Outcome, Context) framework ^45^ which facilitates structuring research questions and defining criteria, crucial for capturing the full scope of the evidence and understanding complex phenomena ^46^.

The criteria in Table 1 were generated after developing the CPTs and will be refined as the review progresses. This iterative approach will ensure a thorough literature exploration, progressively refining the intervention's theoretical framework. This process allows ongoing refinement based on emerging evidence and expert feedback ^12, 28^. The search strategy will facilitate the identification of specific mechanisms and outcomes, enhancing the rigour and depth of the realist review.

**Table 1.** PICOC used to clarify inclusion and exclusion criteria.

PICOC Element	Key concept	Inclusion and exclusion criteria
**Population**	Leaders in healthcare organisations (acute hospitals, systems) who are at a mature stage of implementing Lean.	**Included**: Healthcare organisations (acute hospitals) hospital systems, that have adopted Lean healthcare improvement interventions to a mature stage characterised by sustained improvement, strong leadership commitment, established daily management systems, data-driven decision-making, staff engagement, and empowerment. This stage is typically reached in 5–10 years after initial implementation ^47^ **Excluded**: Healthcare organisations that do not meet this inclusion criterion. **Excluded**: Non-acute healthcare organisations that do not meet this inclusion criterion (Primary Care, mental health specialist services).
Interventions	Lean leadership (behaviors, attributes, values, principles)	**Included**: Leading lean improvement interventions **Excluded**: Other quality improvement interventions
Comparison	Other QI methodologies	**Included**: Lean methodology. **Excluded**: Other QI methodologies. **For consideration**: Whether other QI methodologies mentioned as comparators in the retrieved literature (e.g., combined use of Lean and Six Sigma) will be included where relevant to the research question.
Outcomes	Sustained/ successful implementation adoption of Lean. Culture of continuous improvement.	**Included**: The study will look for evidence of how leadership attributes behaviours, values, and principles have contributed or not contributed to the sustainable implementation of Lean improvement in these settings. **Included**: The study will look for evidence of how leaders in these settings have supported lean implementation by demonstrating key behaviours.
Context	Time Language Setting	The time frame for the consideration of studies will be from 2000 to 2026. Healthcare organisations, organisations delivering services to hospitals. English Acute Hospitals/ acute health systems

***3. Review primary studies and extract data***

Documents retrieved will be selected for their relevance to the research question ^47^ and their contribution to the development of IPTs ^25, 28^. Small sections of a primary study can be relevant when testing specific hypotheses about context, mechanisms, and outcomes ^48^. These documents will form a robust collection of papers, essential for a theory-driven realist review to further refine the IPTs ^49^.

The selection of studies hinges on relevance and rigour. Relevance is assessed by whether a study can contribute to building or testing a programme theory, while rigour refers to the credibility of the methods used to collect the data ^34, 50^. These criteria will guide the inclusion or exclusion of literature in the review ^51^. Data extraction involves three steps: initial screening by title and abstract, full-text retrieval, and appraisal. Titles and abstracts will be reviewed in duplicate by Authors 1 and 2 using predefined criteria ( Table 1). Documents passing this stage will be assessed for richness and rigour by Author 3. Discrepancies will be resolved through discussion among all three authors, adhering to RAMESES guidelines ^34^. To facilitate data extraction, bespoke forms will be developed, tailored to the theoretical framework of the review ^52^. Standardised forms may be unsuitable; hence, tailored forms will capture contextual factors, mechanisms, and outcomes relevant to the research question ^53^.

***4. Synthesize evidence and develop conclusions***

After data extraction, papers will be imported into NVivo ^54^, version 14 (Mac) a specialised qualitative software widely used in realist reviews for coding and thematic analysis to identify Context-Mechanism-Outcome Configurations (CMOCs) which is available freely to students and faculty within UCD. Leaders from healthcare organisations at the forefront of Lean deployment will be re-engaged to refine the theoretical framework, contributing their expertise. Review stages will be revisited as needed to ensure comprehensive data collection and achieve theory saturation. Refined initial program theories (IPTs) from the realist review will then be presented to an expert panel for adjudication and feedback, ensuring robustness, contextual sensitivity, and reflection of intervention complexities ^34, 52^.

***5. Refine theory iteratively and disseminate findings***

The refined Initial Programme Theories (IPTs) derived from the realist review will undergo iterative evaluation and feedback from the expert panel to ensure accurate interpretation of results ^25^. This engagement with the expert panel aims to enhance the robustness, contextual sensitivity, and comprehensive reflection of the inherent complexities in the intervention's implementation and outcomes ^35^.

The outcomes of this realist review will follow RAMESES reporting guidelines ^53^ and will be disseminated through peer-reviewed journal publications, conference presentations to healthcare and academic audiences, and engagement with key stakeholders. Additionally, findings will be integrated into a PhD thesis. This review sets the foundation for a subsequent realist evaluation involving healthcare leaders experienced in Lean implementation whose organisation are at a mature stage of Lean deployment. This realist review protocol is undergoing review by the PROSPERO Editorial committee and we will provide a protocol registration number when this is completed.

### Ethics

Ethical approval is not needed because this study is a synthesis of published literature and no data collection will take place.

## Conclusion

The realist review outlined in this protocol aims to explore Lean leadership within the healthcare sector, investigating its role in addressing the challenges of sustainably deploying Lean methodologies in acute healthcare organisations. Existing literature highlights the importance of effective leadership in sustaining Lean initiatives, fostering continuous improvement, and achieving operational excellence ^1, 19, 20^. Realist inquiry is ideal for this investigation as it comprehensively explores contextual factors, causal mechanisms, and outcomes related to Lean leadership in healthcare ^55^.

This approach identifies not only effective strategies but also the underlying reasons and conditions influencing their success, providing a robust framework for understanding causal mechanisms in complex organisational contexts ^40^. It seeks detailed insights into the attributes and behaviours that characterise effective Lean leadership, significantly contributing to the scholarly understanding of Lean leadership in healthcare ^19^. The study aims to offer practical implications for healthcare management and leadership development, enhancing knowledge of how Lean leadership-attributes, values, principles and behaviours can improve patient and staff experiences, patient outcomes, and organisational performance ^20^. Ultimately, this research will inform policy and practice, ensuring effective implementation and sustainability of Lean leadership strategies in healthcare settings.

## Systematic review registration

This protocol is currently undergoing review by the PROSPERO Editorial committee and registration number will be added as soon as available.

## Ethics

Ethical approval is not needed because this study is a synthesis of published literature and no data collection will take place.

## Consent to participate

This is a protocol paper so not applicable.

## Data Availability

No data are associated with this article Figshare: Whether, to what extent, and in what circumstances and ways do leaders’ and managers’ attributes, values, principles and behaviours contribute to the sustainable implementation of Lean in healthcare. Teeling, Sean Paul (2024). Prisma -P checklist for Realist Review Protocol. figshare. Dataset. https://doi.org/10.6084/m9.figshare.26212916 ^56^ Data are available under the terms of the Creative Commons Attribution 4.0 International license (CC-BY 4.0) Teeling, Sean Paul (2024). Additional data File 1 CPT.docx. figshare. Dataset. https://doi.org/10.6084/m9.figshare.26212862.v3 ^57^ Data are available under the terms of the Creative Commons Attribution 4.0 International license (CC-BY 4.0)
