# Supplementary material for: The contribution of leaders' and managers' attributes, values, principles, and behaviours to the sustainable implementation of Lean in healthcare: A realist review protocol
Source: HRB Open Res. 2024 Aug 13;7:54. [Version 1] doi: 10.12688/hrbopenres.13933.1 (PMC11786105; doi:10.12688/hrbopenres.13933.1)
Supplement: Peer review history

# Reviewer report: Reviewer response for version 1

**DOI:** 10.21956/hrbopenres.15288.r43324

**Published:** 2025-01-31

**Authors:** Rebecca Hardwick

**Affiliations:** 1 University of Plymouth, Plymouth, UK

This paper sets out a process for surfacing the underlying mechanisms that drive leadership of lean interventions in healthcare. It proposes using a realist review methodology to develop context, mechanism outcome explanations of how sustainability of lean outcomes are achieved by leadership behaviours.

 The study rationale and objectives, and research question are clear and appropriate, and the study design is an excellent choice for interrogating these issues. Whilst I have said that the study design and methods are clearly laid out to allow replication from others, this is not a necessary mark of quality in realist reviews - programme theory building and refining is an interpretative process, and does not follow the usual rules for traditional systematic review in this way - I think the authors know this though, and no changes need to be made. 

 My only query is why the inclusion criteria is focusing on acute hospitals alone - whilst logical in some respects, including other health care (or indeed wider, such as VCSE settings) where Lean has been implemented may yield very useful information to further refine programme theory: my question is, what else might be learnt from including other settings?  In realist work, context is more than 'place' or geography, but is really about that which supports or inhibits the mechanisms 'firing' - including studies from other settings may provide useful insights to refine the CPTs.

Is the study design appropriate for the research question?

Yes

Is the rationale for, and objectives of, the study clearly described?

Yes

Are sufficient details of the methods provided to allow replication by others?

Yes

Are the datasets clearly presented in a useable and accessible format?

Not applicable

Reviewer Expertise:

Applied health and care services research in mental health, realist methodology, and knowledge mobilisation.

I confirm that I have read this submission and believe that I have an appropriate level of expertise to confirm that it is of an acceptable scientific standard.

# Reviewer report: Reviewer response for version 1

**DOI:** 10.21956/hrbopenres.15288.r43326

**Published:** 2024-11-29

**Authors:** Joy Furnival

**Affiliations:** 1 The University of Manchester, Manchester, England, UK; 2 Care Quality Commission, London, UK

This protocol is well structured and written and there is a clear and compelling argument for the study.

 Whilst this is a good protocol, 2 areas could strengthen this further.  1) how will learning about Lean leadership from other sectors and/or healthcare, or with text books/academic chapters rather than papers/websites/grey literature be incorporated if needed 2) The inclusion criteria on Lean maturity for incorporation within the study is quite strong, given the low numbers of Lean healthcare organisations reaching this level of commitment - only 12% cited in the US study, and much may not be written into the research literature, this criteria may need further review to ensure a sufficiently meaningful sample size following the protocol.

Is the study design appropriate for the research question?

Yes

Is the rationale for, and objectives of, the study clearly described?

Yes

Are sufficient details of the methods provided to allow replication by others?

Yes

Are the datasets clearly presented in a useable and accessible format?

Yes

Reviewer Expertise:

Quality improvement and regulation in healthcare

I confirm that I have read this submission and believe that I have an appropriate level of expertise to confirm that it is of an acceptable scientific standard.
